# Review of Dr. N. S. Davis’ Annual Address before the Illinois State Medical Society

**Published:** 1857-01

**Authors:** 


From the Western Lancet.

*Review of Dr. N. S. Davis' Annual Address before the Illinois State Medical Society, read at the Annual Meeting in Vandalia, June* 4, 1856.

The address of Dr. Davis is designed to show the evil effects of *alcohol*, in that state of the system known as *tuberculosis.* The author admits the general view to be correct which assigns to the tuberculous diathesis a low grade of nutritive actions; but he denies, in the most emphatic manner, that alcohol, or any of its preparations, can in any manner counteract the morbid conditions found either in the predisposition, or the openly developed disease. The following extract will give a clear conception of his want of faith in the virtues of alcohol:

"Some of my audience may be ready to ask, if I would deny the almost universal sentiment of mankind, by claiming the broad ground that alcoholic liquors are neither tonic, invigorating, or supporting to the human system? I answer, unhesitatingly, yes! and base my answer not merely on the deductions of the most careful and varied experimental researches, but ask, in return, what there is in the tottering and unsteady step, the impaired digestion, the frequent functional derangements of the kidneys, exhibited in a greater or less degree by all habitual drinkers, which is indicative of increased physical strength, vigor, or power of endurance?"

It appears from the above extract, that the author denies to spirituous compounds either *tonic, invigorating or supporting effects*? Surely Dr. D. must have been laboring under some singular spirit of philosophical hallucination when he reached this conclusion. What will the surgeons think, who have administered these agents to patients laboring under excessive suppuration, gangrene, and other states of debility; or what the physicians, who have followed Stokes in the administration of wine in typhus fever, when the first sound of the heart becomes feeble? We can ourselves look back with surprise to an instance in which forty-eight pints of wine were administered to a typhoid fever patient! We thought at the time that the wine saved the patient's life; but if we can trust the conclusions of Dr. Davis, we were administering to him an agent which was not only entirely devoid of tonic, invigorating, or supporting influence, but in fact progressively debilitating and prostrating the system. Still, we have the satisfaction of remembering that the patient finally recovered from what seemed an almost hopeless state of prostration. We will leave Dr. Davis to explain how this could have happened, if wine possesses no "tonic, invigorating, or supporting" effect!

But Dr. Davis proceeds to explain why alcoholic preparations are pernicious in tuberculosis, either as a prophylactic or curative agent. The objections to alcoholic stimulus are the following: It diminishes the excretions, especially of carbonic acid, retards the conversion of venous into arterial blood, lessens the organic changes, depresses the temperature of the body, and lessens the tone of the muscular structures. Let us inquire more carefully into these effects.

1. *Alcoholic preparations diminish the exhalation of carbonic acid by the lungs.* This proposition has been clearly proven by many careful observers and experimenters. The explanation offered by Liebig, that the alcoholic preparations contain but a small proportion of carbon, with an excess of hydrogen, and that the latter is really the heat-producing agent, may have some force. It is certainly true, that the watery exhalation from the lungs, when alcohol has been taken, is not diminished; and hence it may be inferred, that the oxygen combines with the hydrogen, while the carbon remains unchanged. In this way a very rapid accumulation of carbon would take place in the blood, and the oxygen being appropriated to the hydrogen of the alcohol, the venous blood would finally predominate, *provided the metamorphosis of the tissues furnish the usual proportion.*

2 *It depresses the temperature of the body.* We cannot assent to this proposition, except in a conditional sense. If alcohol unconditionally diminishes animal heat, then we could safely and beneficially employ it in cases of inflammation, or any disease attended by febrile heat. So far, however, from this being recognized as a correct course of practice, we presume a physician who would propose such a measure, would be regarded as demented.

The truth is, the heat-producing power of alcohol is altogether *conditional.* If given to a person in ordinary health, and in considerable quantities, so as to raise the carbon of the blood above the physiological condition, the depressing effects, at least secondarily, will finally be felt. The first and direct effect will be that of excitation ; the second, that of super-carbonization and depression of the nervous system. It will follow, therefore, that animal heat will be diminished in this secondary condition, as a result of the depression which always follows over-stimulation, and also from the presence of a superabundance of carbon.

This is one point of view ; let us examine the opposite. Suppose the system to be greatly emaciated—the tissues reduced to the lowest state of decomposition, and the carbon or heat-producing agent, almost exhausted. What then would be the effect of alcoholic preparations ? Surely a *moderate* quantity of hydro-carbon would supply, to some extent, the deficiency, and the oxygen acting on this dement, would, beyond all doubt, augment animal heat. It will be perceived, therefore, that in one example alcohol will become a disease-producing agent, and diminish animal heat; while in the opposite condition, it will supply hydro-carbon, and thereby contribute to the development of caloric.

3. *It diminishes organic changes.* This is evidently correct ; alcoholic preparations diminish the process of healthy metamorphosis, in a physiological state of the system, *and it is equally efficient in arresting emaciation in morbid conditions.* Now, while we would have no desire to secure the first condition (that is, to fatten a *healthy man* with spirituous potations), it might become exceedingly important to arrest progressive emaciation, such as occurs in phthisis, by the administration of these stimulants.

We think it is abundantly evident that Dr. Davis' premises are false, and therefore his conclusions vitiated. His great error consists in assuming that the morbid effects of large quantities of spirits, on a healthy system, can be taken as evidence of the therapeutical action of duly proportioned quantities in morbid conditions requiring stimulants. We have not before us the record of the experiments performed by Dr. Davis ; but the following extract from the Address conveys a clear idea of the kind of facts on which he relies. In support of the opinion that alcoholic preparations cause a retention of carbon in the system, he says :

" So marked, indeed, is this effect that, in some of my experiments, the air exhaled from the lungs one hour after three or four ounces of brandy had been taken into the stomach, contained more than twenty-five per cent. less than the normal proportion of carbonic acid gas. And whoever will observe the purple lips, the bloated and leaden face, the slow and almost stertorous breathing of full intoxication, will see abundant proof of impeded circulation through the pulmonary capillaries."

It is abundantly evident from the above extract, as well as the tenor of the whole address before us, that the author has committed the common error of drawing his conclusions from the *pathological* instead of the *therapeutical* effects of alcohol. The introduction of " three or four ounces" of brandy into the stomach certainly far exceeds an ordinary therapeutical dose ; and the " purple lips, the bloated, leaden face, the slow and stertorous breathing of full intoxication," are simply evidences of the morbid effects of excessive quantities. What would Dr. Davis think of our philosophy if we should denounce opium as unqualifiedly injurious, and appeal to the diminished sensation, stertorous breathing, and even convulsions, in proof of our assumptions? And yet there would be as much propriety and force in such reasoning, as that which he has seen proper to adopt.

In the experiments of Dr. Bocker (which are among the most careful and valuable made on this subject), he took only a *tea-spoonful* of spirit of wine; while Dr. Davis attempts to draw conclusions from the effects of "three or four ounces" of brandy taken at once, or even from full intoxication! Surely no course of experiment or reasoning could be more fallacious and unphilosophical.

If any other evidence could be required to show the difference between the moderate and immoderate (therapeutical and pathological) effects of alcohol, we may refer to the effects produced on the blood. Habitual and excessive use of alcoholic preparations (*alcoholismus*) diminishes the solid contituents of the blood, reduces the proportion of red corpuscles, and impairs the condition of the fibrin with a superabundance of fatty matter. On the contrary, it has been shown that after a moderate use of beer, the watery proportion of the blood was diminished, while the fibrin and red corpuscles were increased. In short, the excessive use of alcohol impoverishes and depraves the blood, and speedily induces disease; while the judicious employment of these preparations may be made to invigorate the system, and promote assimilation.

An obvious and important error in Dr. Davis' theory is, that he denies to alcoholic preparations any tonic, invigorating or supporting power. If the author of the address will look at the rubicund countenance, red nose, florid complexion, and active circulation of the dram-drinker (before disease supervenes), he will hardly deny that alcoholic preparations possess very decided and potent stimulating and sustaining properties. The author will probably find that he has mistaken the temporary depression following over-stimulation, for the legitimate and necessary results of medicinal portions of distilled or malt liquors.

But there is another and even more important error involved in this broad and sweeping denial, namely, the failure to recognize the stimulating effects of these agents on the *digestive system.* All observations abundantly prove that, in debilitated states of the system (free from gastric irritation), alcoholic preparations improve the appetite, give greater force to the function of digestion, and therefore increase the tone of the body in a general sense. It is this way, at least in part, that these preparations become valuable in certain cases of tuberculosis. All must admit that it is an essential point in pulmonary consumption, to maintain primary digestion in as healthy and vigorous a condition as possible; and we believe candid experience will prove that no mere *drug* will do this so well, as a general rule, as the proper administration of alcoholic stimulants. Whatever difference of opinion may exist in regard to the effects of retained carbon, no candid and accurate observer can doubt the beneficial effects of alcoholic stimulants, when given in suitable cases, and proper forms and doses, in promoting primary assimilation.

The second part of the address is devoted to the detail of cases to prove, clinically, that alcoholic preparations produce no beneficial effect in tuberculosis. The author made observations in 37 cases, 24 of whom were natives of Ireland. Of the whole number only 6 were teetotalers, while 31 drank more or less alcoholic liquors.

We are not able to perceive that these cases prove any thing in the premises. It will be remarked that a majority of the patients were Irish; and with the well-known habits of a majority of such persons who are usually ill fed and much exposed, it is not surprising that the alcoholic liquors should prove ineffectual. Nay, more, it is quite probable, if not positively certain, that the want of substantial food, will under the effects of the liquor be positively pernicious. Most of the persons drink liquors of the worst possible quality, and in an irregular manner; often excessively, so that great evils will usually follow.

We might oppose to this kind of evidence our own experience and that of many members of the profession. Thus we have known a case in which an habitual drinker, who died from accidental disease, exhibited evidences *post mortem* of a cicatrized tuberculous cavity. We might appeal, also, to numerous instances in which fermented and distilled liquors, conjoined with other agents, proved eminently useful. Thus we would leave the clinical observations of Dr. Davis and ourselves just where we find them: viz., undetermined. It will require very extended and accurate observations to determine the exact therapeutical value of alcohol in tuberculosis, and the question is evidently yet undetermined; but when we witness a steady improvement of appetite, an arrest or diminution of emaciation, an increase of weight and strength, we cannot avoid the conclusion that agents which produce these effects must be capable of fulfilling at least some of the indications in treatment.

We desire now to define our own position, otherwise very erroneous conclusions might be drawn from the preceding observations. In the first place, we do not advocate the indiscriminate, much less the excessive, use of alcoholic liquors in tuberculosis; and, again, we never employ it as the *principal* agent. We believe, however, that it promotes primary digestion, and thereby invigorates the general system; that it checks excessive transformation of the tissues, and furnishes materials for sustaining animal heat; that it produces a certain amount of nervous excitement which, within proper limits, serves to sustain the vital actions. This is the extent to which we employ it, never allowing excessive stimulation, and never permitting it to be taken except in conjunction with nutritious aliment.

In closing these hasty remarks we feel impelled to make a single observation on the subject of *teetotalism*. We believe that alcoholic liquors are unnecessary in a state of perfect health; that they do nothing to sustain the system under protracted labor, or exposure to cold. The only exception to this last rule is, that when the food has been greatly reduced, moderate quantities of spirits may seem to supply hydro-carbon, and protect the system from rapid emaciation and increasing cold. We subscribe, therefore, mainly, to the teetotal system; but we never permit these opinions to influence, in the slightest degree, the use of alcoholic preparations as medicines. No fear of making our patient a drunkard can deter us from giving such a remedy as his exigencies seem to require. If we permit certain moral abstractions to influence our practice, we would soon be left with a most meagre materia medica. Opium, on this principle, must likewise be banished under the apprehension that we may make our patient an opium-eater. There is but little fear, indeed, of making patients drunkards when their stimulants are given *with* and *as* medicines; but whatever may be the result, medical science rests on too broad a basis to be circumscribed by the dogmas and visionary abstractions of ultraism or ultraists.

In conclusion, we beg to assure Dr. Davis that our opinions have been expressed with candor and with the highest respect to him individually, but with a firm conviction that he is occupying false and untenable ground, and that he has been led into the position more as the result of ultra temperance notions, than calm and philosophic investigations. We beg him to review the subject, and we sincerely believe he will finally reach very different and more wholesome conclusions. L. M. L.

